# Cholecystocutaneous fistula after cholecystectomy

**DOI:** 10.1093/jscr/rjae617

**Published:** 2024-10-04

**Authors:** Raju Sah, Sushil Bahadur Rawal, Srijan Malla, Jyoti Rayamajhi, Pawan Singh Bhat

**Affiliations:** Surgical Gastroenterology Department, Nepal Mediciti Hospital, Karyabinayak, Lalitpur 44600, Nepal; Surgical Gastroenterology Department, Nepal Mediciti Hospital, Karyabinayak, Lalitpur 44600, Nepal; Surgical Gastroenterology Department, Nepal Mediciti Hospital, Karyabinayak, Lalitpur 44600, Nepal; Surgical Gastroenterology Department, Nepal Mediciti Hospital, Karyabinayak, Lalitpur 44600, Nepal; Surgical Gastroenterology Department, Nepal Mediciti Hospital, Karyabinayak, Lalitpur 44600, Nepal

**Keywords:** cholecystocutaneous fistula, open cholecystectomy, cholecystitis

## Abstract

Cholecystocutaneous fistula is an exceedingly rare type of external biliary fistula, where an abnormal connection forms between the gallbladder and the skin. Cholecystocutaneous fistula commonly develops in the setting of chronic calculus cholecystitis or following a previous surgical intervention involving the biliary tract. Patients with cholecystocutaneous fistula often present with systemic symptoms, such as fever, nausea, and vomiting, as well as localized symptoms in the right upper quadrant of the abdomen, where the external opening of the fistula is typically found. Ultrasonography, computed tomography, magnetic resonance imaging, magnetic resonance cholangiopancreatography (MRCP), and fistulograms (computed tomography or X-ray) are commonly used. Computed tomography has proven to be more effective than ultrasonography in delineating the fistulous tract and the associated fluid collections. Open cholecystectomy with excision of the fistulous tract is considered the gold standard and is curative in the majority of cases. However, a laparoscopic approach has become a viable alternative, especially in the hands of experienced surgeons.

## Introduction

Fistula is an abnormal condition resulting from an abnormal connection between two epithelialized surfaces. Biliary fistulas are rare complications of gallstones that connect the biliary tract to other organs, categorized as either external or internal [1]. Internal biliary fistulas connect the gallbladder with the gastrointestinal tract, typically induced by chronic cholecystitis [2]. External biliary fistulas connect the gallbladder with the abdominal wall and can be spontaneous, postoperative, post-traumatic, or caused by iatrogenic injury. Cholecystocutaneous fistula (CCF) is a type of external biliary fistula connecting the gallbladder with the skin [1, 3].

## Case report

A 35-year-old male presented to our outpatient department with complaints of swelling, pain, and discharge from the right upper quadrant of the abdomen for 2 years. He had no history of fever, nausea, vomiting, or medical co-morbidities. He had undergone a laparoscopic cholecystectomy converted to open surgery two and a half years prior and excision of a sinus tract one and a half years prior.

**Figure 1:**
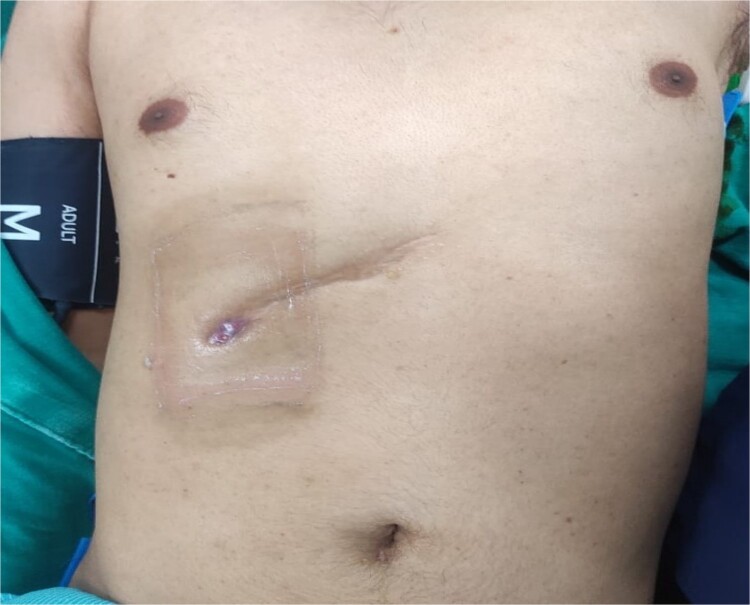
Showing Sinus opening at previous operated site over right subcostal region.

Physical examination revealed a conscious, cooperative, and well-oriented patient with normal vital signs. Local examination showed a discharging sinus in the right upper abdomen through an old surgical scar, with pus flowing upon pressure (Fig. 1). Blood investigation revealed a total white cell count of 14 000.

Ultrasound of the abdomen and pelvis showed an echogenic focus measuring 13.1 mm, likely a calculus in the superior aspect of the gallbladder fossa, with a surrounding collection measuring 17 × 15.3 mm, likely an abscess, forming an elongated sinus tract extending to the external skin of the scar site.

A CT scan of the abdomen revealed a walled-off fluid collection ~5.1 × 1.9 × 1.6 cm in the gallbladder fossa, penetrating segment 4a of the liver, with a small calcific density attached to the wall of this fluid collection. A soft tissue density tract-like lesion extended from the anterior aspect of the collection toward the subcutaneous plane of the right upper abdominal wall, suggesting a sinus tract (Fig. 3).

**Figure 2:**
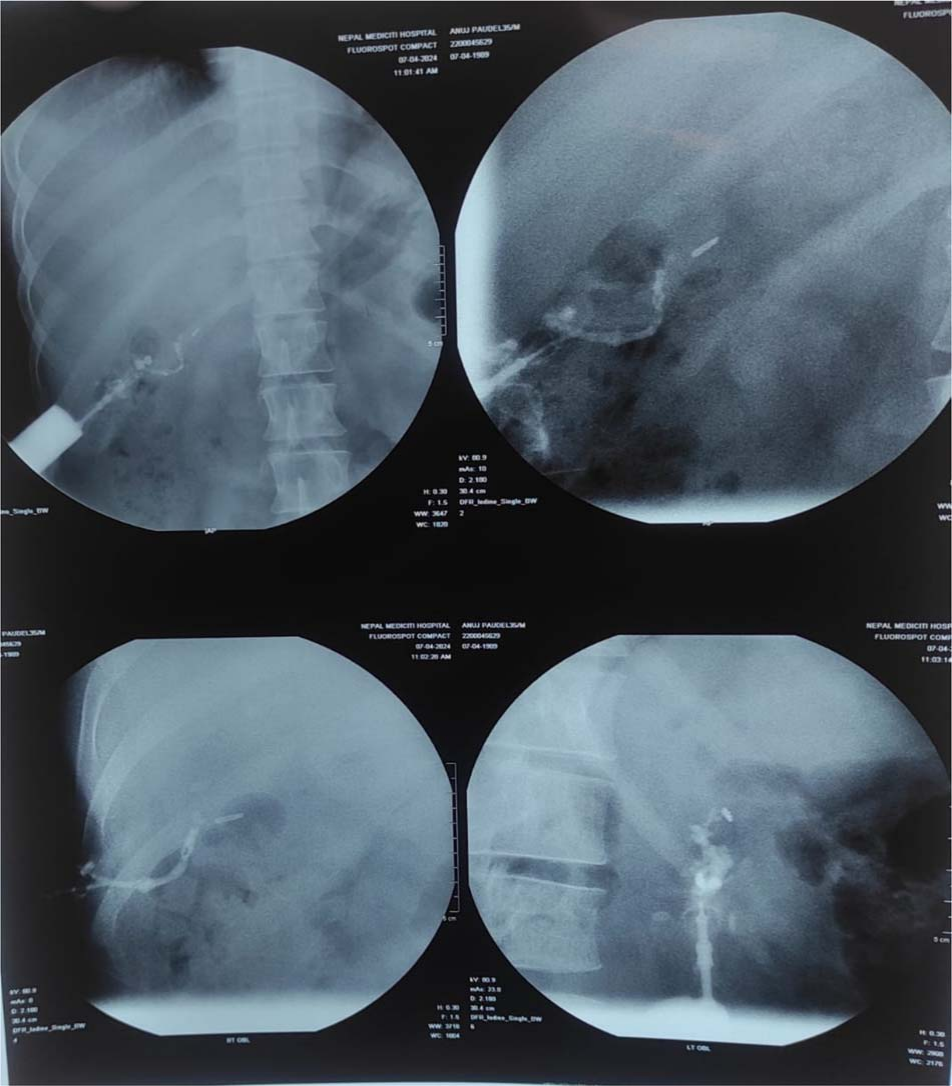
Sinogram showing blind ending tract extending from sinus opening upto the surgical clips in GB fossa probably biliarycutaneous fistula.

Sinogram showed a blind-ending tube up to the surgical clips in the gallbladder fossa, representing a biliary-cutaneous fistula (Fig. 2).

**Figure 3:**
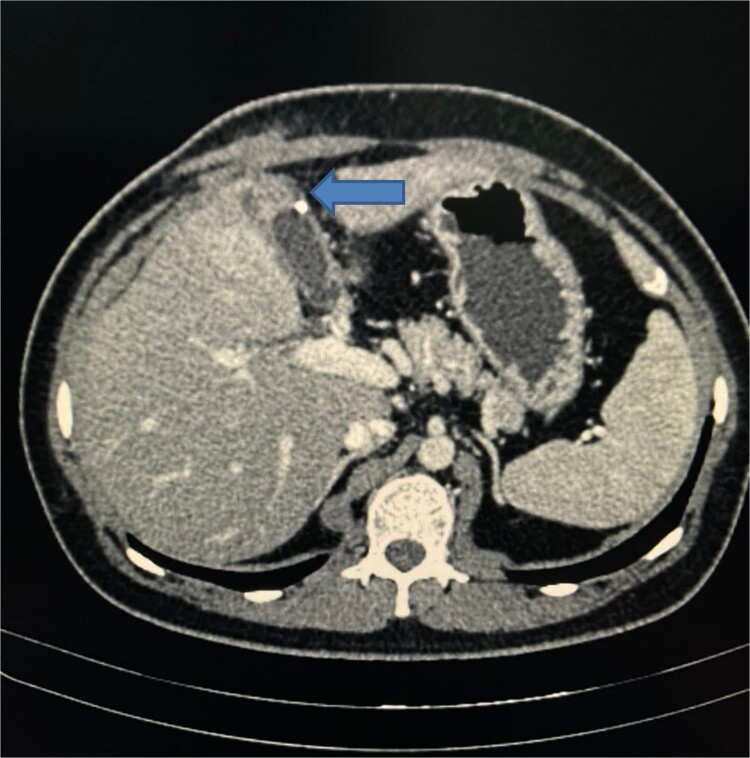
CT scan of abdomen showing a walled-off fluid collection with a small calcific density and a soft tissue density tract-like lesion extended from the anterior aspect of the collection towards the subcutaneous plane of the right upper abdominal wall, suggesting a sinus tract.

**Figure 4:**
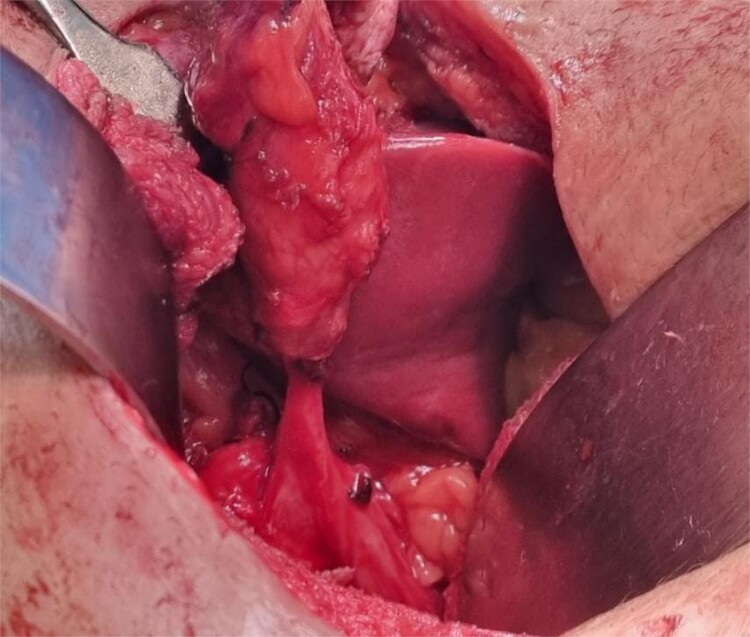
Intraoperative image showing cystocutaneous fistula tract with remnant gall bladder.

**Figure 5:**
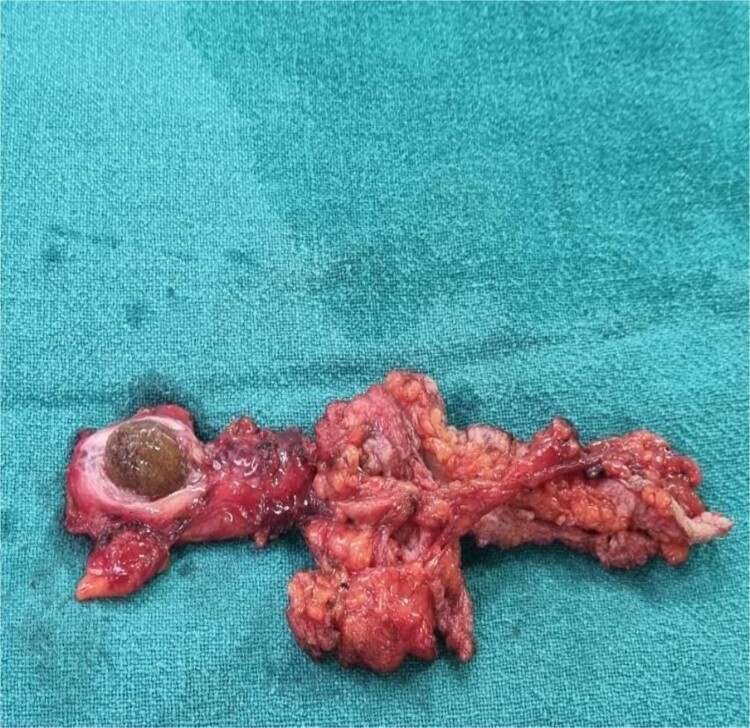
Intraoperative image showing cystocutaneous fistula tract with stone in remnant gall bladder

## Diagnosis and treatment

The diagnosis of CCF post-cholecystectomy was made based on history and examination. Exploratory laparotomy was performed after routine preoperative examination. Intraoperatively, a residual gallbladder or cystic duct with stone was found, ~3 × 3 cm in size, adhered to surrounding tissues and parts of the intestinal walls (Figs 4 and 5). The intact fistula was dissected out, and the adhesion tissue around the gallbladder or cystic duct was separated. The cystic duct was identified, confirmed, clamped, and removed along with the residual gallbladder or cystic duct with stone. Postoperative antibiotics and supportive treatment were administered, and the patient was discharged on the second postoperative day.

## Discussion

Biliary fistulas can be either internal or external. Internal fistulas are more common, with 75% connecting to the duodenum and 15% to the colon. The remaining 10% connect to the stomach, jejunum, or have multiple communications, such as cholecystoduodenocolic fistula [4].

External biliary fistulas are rare. They commonly present with chronic calculus cholecystitis or a history of previous surgical intervention, such as subtotal cholecystectomy for acute cholecystitis. Most cases of CCF are related to bacterial infection in the gallbladder, but some arise from gallbladder adenocarcinoma. *Escherichia coli* is the most common microorganism found in CCF cases, followed by coliforms and Klebsiella pneumonia. Retained stones after laparoscopic cholecystectomy and traumatic rupture of the gallbladder are also predisposing factors for CCF [5].

Biliary outflow obstruction increases intramural pressure, restricts gallbladder perfusion, and precipitates necrosis and perforation of the gallbladder. Once perforated, it may drain into the peritoneal cavity, adjacent viscera, or less commonly adhere to the abdominal wall to form an external fistula [6].

The external opening of a CCF is generally in the right hypochondrium, but other sites can be involved, such as the left hypochondrium (45%), the umbilicus (27%), the right lumbar region, the right iliac fossa, and the gluteal region [4].

The general condition of the patient varies based on age and past medical history. There is usually a history of gallbladder calculi or neglected gallbladder disease. CCF patients present with systemic symptoms like fever, nausea, vomiting, pain, swelling in the RUQ or epigastric region, discharging sinus from the anterior abdominal wall, erythematous mass, or subcostal abscess [5].

Chronic osteomyelitis of the ribs, discharging tuberculoma, infected epidermal inclusion cyst, metastatic carcinoma, and pyogenic granuloma are potential diagnoses that must be considered and ruled out [7]. Infections involving the ribs, especially when associated with a history of gallbladder disease, can present with draining sinuses and can be confused with a fistula [8]. Gallbladder or biliary tree malignancies may present with fistulas, but they are often associated with other systemic signs like weight loss, jaundice, and palpable mass [9]. Inflammatory bowel disease can cause fistulas, including entero-cutaneous fistulas that may resemble a CCF if located near the gallbladder [10].

CCF is diagnosed using imaging studies or exploratory laparotomy in special cases. Imaging studies include ultrasonography (US), computed tomography (CT), fistulogram, and magnetic resonance imaging (MRI) [5].

Management of a CCF involves broad-spectrum antibiotics, incision, and drainage of the sinus abscess, and sending samples for culture and sensitivity. After the acute phase, elective cholecystectomy and excision of the fistula are performed, usually by open surgery [11].

Open cholecystectomy with excision of the fistulous tract is considered the standard management option and is curative in most cases. However, laparoscopic cholecystectomy with excision of the tract is an acceptable and preferable option for experienced laparoscopic surgeons [5].

Interventional radiology (IR) can be crucial in managing CCFs, particularly for patients who cannot undergo surgery or when the fistula presents with complex anatomy or infection. Treatment options include percutaneous drainage, cholecystostomy, biliary stenting, ablation techniques, and imaging-guided embolization of the fistula tract [12–14].

## Conclusion

CCF is a rare condition. Diagnosis requires a high index of suspicion, especially in patients with a history of cholecystitis or previous cholecystectomy. CCF should be considered as a differential diagnosis in patients presenting with persistent abdominal pain, discharge from the abdominal wall, or other signs of biliary tract pathology. Open cholecystectomy with excision of the fistulous tract is considered the standard management option and is curative in most cases.

## Conflict of interest statement

None declared.

## Funding

No financial support or benefits have been received by any of the authors for this study.
